# Evaluation of the predictive accuracy of QUICKI and McAuley indices for insulin resistance in adolescents: Insights from a cross-sectional study

**DOI:** 10.1371/journal.pone.0347304

**Published:** 2026-04-17

**Authors:** Miriam Mohatar-Barba, Ángel Fernández-Aparicio, Javier S. Perona, Jacqueline Schmidt-RioValle, Carmen Enrique-Mirón, Emilio González-Jiménez

**Affiliations:** 1 Department of Nursing, Faculty of Health Sciences, Melilla Campus, University of Granada, Melilla, Spain; 2 Instituto de Investigación Biosanitaria (ibs.GRANADA), Granada, Spain; 3 Department of Nursing, Faculty of Health Sciences, University of Granada, Granada, Spain; 4 Department of Food and Health, Instituto de la Grasa-CSIC, Campus of the University Pablo de Olavide, Seville, Spain; 5 Department of Inorganic Chemistry, HUM-613 Research Group, Faculty of Health Sciences, Melilla Campus, University of Granada, Melilla, Spain; Instituto Mexicano del Seguro Social, MEXICO

## Abstract

Different indirect methods have been developed to assess insulin resistance (IR), though their validation has been limited to adult populations. In this sense, the study aim is to compare the predictive capacity of the McAuley, QUICKI, SPISE indices, and glucose-insulin ratio against insulin resistance (IR) in Spanish adolescents and to establish reliable cut-off values for these indices in this population. A cross-sectional study was conducted with 981 adolescents aged 11–16 years, from Southern Spain. Anthropometric measurements and fasting biochemical parameters, were assessed. IR indices, such as HOMA-IR, QUICKI, the McAuley index, SPISE, and the glucose-insulin ratio, were calculated. The ability of each index to predict IR was evaluated using multivariate regression analysis and receiver operating characteristic (ROC) curves. Boys exhibited higher waist circumference, triglyceride levels, and fasting insulin levels, while girls had a higher percentage of body fat (p < 0.05). HOMA-IR values increased in adolescents with obesity, whereas McAuley and QUICKI indices decreased significantly (p < 0.05). Similar results were found in adolescents with MetS in comparison to non-MetS adolescents. QUICKI showed the highest predictive accuracy (AUC: 1.00), followed by the McAuley index (AUC: 0.983 in boys, 0.977 in girls), and SPISE (AUC: 0.896 in boys, 0.852 in girls). The cut-off points for these indices were 5.794,0.316, and 7.8 respectively, both for boys and girls. The glucose-insulin ratio demonstrated poor predictive ability. The QUICKI, McAuley, and SPISE indices are effective in predicting IR among Spanish adolescents. Their use could aid in early detection of metabolic and cardiovascular disfunctions, though further validation in larger cohorts is required for clinical application.

## Introduction

The rising prevalence of obesity in adolescents worldwide is contributing to an increased incidence of insulin resistance (IR) and type 2 diabetes [1]. This trend is particularly concerning, as IR plays a key role in the pathophysiology of metabolic syndrome (MetS) [2]. Moreover, a higher degree of IR in children and adolescents has been linked to the early clustering of cardiovascular risk factors, indicating a greater likelihood of future cardiovascular disease in this population [3–5].

Accurate tools for assessing insulin sensitivity in adolescents at risk are therefore vital to determine the presence and severity of IR [6–8]. Various methods exist to evaluate IR, both direct and indirect. Among the direct techniques, the euglycemic-hyperinsulinemic clamp (EHC) remains the reference standard. Despite its precision, its complexity, time requirements, and cost limit its use, particularly in large-scale studies [9].

Consequently, indirect and non-invasive approaches have become more widespread in clinical and epidemiological contexts [6,10,11]. One of the most frequently used methods is the Homeostatic Model Assessment for Insulin Resistance (HOMA-IR), which relies on fasting plasma insulin and glucose levels and has been validated in both adolescent and adult populations [12–14].

In recent years, additional surrogate indices such as the McAuley index, the Quantitative Insulin Sensitivity Check Index (QUICKI), and the glucose-to-insulin ratio have been proposed as alternatives to HOMA-IR for estimating IR [15–18]. The McAuley index incorporates fasting insulin and triglyceride levels and has demonstrated strong correlation with the EHC, particularly in normoglycemic individuals [19]. Similarly, QUICKI has shown a robust association with insulin sensitivity and may outperform HOMA-IR in predicting type 2 diabetes risk [20,21]. These indices, along with the glucose-to-insulin ratio, have proven useful in adult populations and epidemiological settings, but their utility in adolescents remains underexplored.

Given this background, the present study aimed to evaluate and compare the predictive value of the McAuley, QUICKI, and SPISE indices, as well as the glucose-to-insulin ratio, for identifying IR in adolescents. The ultimate goal was to establish cut-off thresholds that could support early detection of IR in this age group.

## Methodology

### Study design and sample

A cross-sectional study was conducted with 981 adolescents (456 boys and 525 girls), aged 11–16 years, enrolled in 18 public and private schools located in urban and rural areas of the provinces of Granada (Andalusia, Southern Spain). The recruitment process was conducted among September 2014 to July 2018. To participate in the study, an invitation letter was sent to the principals of the 18 schools, all of whom agreed to participate. From the 18 schools, two classes per grade out of three were randomly selected, and were invited to participate in the study. To be included, participants had to be healthy and free from any metabolic disorders or physical dysfunctions. Adolescents who did not meet these criteria were excluded from the study. The study reports adhere to the general EQUATOR guidelines [22]. The study was approved by the Ethics Committee of the University of Granada (protocol code 841) and authorized by the principals of the participating schools. Additionally, written informed consents were obtained from all parents or legal guardians of the adolescents prior to the beginning of the study, in accordance with the Declaration of Helsinki [23].

### Anthropometric parameters

An anthropometric evaluation was performed on each participant according to the guidelines of the International Society for the Advancement of Kinanthropometry (ISAK) [24]. This assessment was conducted by a Level 2 anthropometrist certified by ISAK. The measurements were taken between 8:30 and 10:30 a.m., following a 12-hour fast and 48 hours of exercise abstention. To ensure the privacy of the participants, all measurements were taken individually in a designated classroom at each school. Body weight (kg) was measured twice (with participants barefoot and in light clothing) using a self-calibrating digital floor scale, model Seca 861 Class III (Saint Paul, USA), with an accuracy of up to 100 g. Height was measured using a Seca stadiometer model 214 (Saint Paul, USA), with an accuracy of 1 cm. Body Mass Index (BMI) was calculated as weight in kilograms divided by height in meters squared (BMI = kg/m²). Waist circumference (WC) was measured using a retractable automatic Seca tape (accuracy of 1 mm) at the horizontal plane midway between the lower rib and the top of the iliac crest at the end of a normal exhalation. Hip circumference (HC) was measured at the widest part around the buttocks. Waist-to-hip ratio (WHR) was calculated by dividing waist measurement by hip measurement (W/H). Skinfolds at the triceps, biceps, subscapular, and suprailiac regions were also measured using a Holtain skinfold caliper (Holtain Ltd., Crymych, United Kingdom), with an accuracy of 0.1–0.2 mm. Body fat percentage was calculated based on skinfold measurements. The Brook equation [25] was initially used to calculate body density, and the body fat percentage was then determined using Siri’s equation [26].

### Biochemical parameters

Venous blood samples were drawn after a 12-hour overnight fast by qualified professionals. At 8:00 a.m., 10 mL of blood were collected via venipuncture from the right arm’s antecubital fossa, using a disposable vacuum blood collection tube. All samples were centrifuged at 1300 g for 15 minutes (Z400 K; Hermle) within 4 hours of collection. Glucose concentration was measured using an enzymatic colorimetric method (glucose oxidase-phenol amino-phenazone (GOD-PAP) method; Human Diagnostics, Wiesbaden, Germany), along with HDL-c, total cholesterol, and triglycerides (TG) concentrations using enzymatic colorimetric methods with an Olympus analyzer (Westborough, MA, USA). LDL cholesterol (LDL-c) was estimated using the Friedewald equation [(LDL-c) = (total cholesterol) – (HDL-c) – ([TG]/5)]. Serum insulin was measured by radioimmunoassay (Insulin Kit; DPC, Los Angeles, USA). Insulin resistance was quantified using the homeostasis model assessment (HOMA) with the following equation: fasting glucose (mmol/L) × fasting insulin (mU/L)/ 22.5.

### Blood pressure measurement

Blood pressure (BP) was measured using a previously calibrated sphygmomanometer and a Littmann® stethoscope, following the recommendations of the Subcommittee of Professional and Public Education of the American Heart Association’s Council on High Blood Pressure Research [27]. Participants were asked to relax and remain silent during BP measurement. A systolic blood pressure ≥ 130 mmHg and/or diastolic blood pressure ≥ 85 mmHg were considered risk factors for MetS.

### Definition of metabolic syndrome

The criteria defined by the International Diabetes Federation (IDF) [28] were used to diagnose MetS. According to these criteria, MetS is diagnosed in adolescents when they have a waist circumference ≥ 94 cm in boys and ≥ 80 cm in girls, along with at least two of the following risk factors: fasting glucose levels between 100–125 mg/dL, serum TG levels ≥ 150 mg/dL, HDL cholesterol (HDL-c) concentrations < 40 mg/dL in boys and < 50 mg/dL in girls, and BP ≥ 130/85 mmHg. Adolescents meeting these criteria were classified as having MetS, while those who did not were classified as non-MetS.

### Insulin resistance indicators

The IR indicators used and calculated in this study were the following:

HOMA-IR was calculated by using the following equation [29]:


$$HOMA-IR=\frac{Fasting\;insulin\;(\mu \text{U}/\text{mL})\times Fasting\;glucose\;(mg/dL)}{\text{4}\text{0}\text{5}}$$

QUICKI index was calculated as follows [30]:


$$QUICKI\;Index=\frac{\text{1}}{\text{log}(Fasting\;insulin(\mu \text{U}/\text{mL}))\;+\text{log}\left(Fasting\;glucose\;(mg/dL\right)}$$

McAuley index was calculated as follows [19]:


McAuley Index=exp[2.63 −0.28×ln(Fasting insulin(μU/mL)) −0.31×ln(TG(mmol/L))]

Glucose to insulin ratio was calculated by dividing fasting glucose (mg/dL) by the fasting insulin (µU/mL) [31].

SPISE index was calculated as follows [32]:


$$SPISE=\;\frac{\text{6}\text{0}\text{0}\times {HDL-C}^{\text{0}.\text{1}\text{8}\text{5}}}{{TG}^{\text{0}.\text{2}}\times {BMI}^{\text{1}.\text{3}\text{3}\text{8}}}$$

### Statistical analysis

All calculations were performed using SPSS v24.0 (IBM, Armonk, New York, USA). A p-value of < 0.05 was considered statistically significant. Normality of the distribution was assessed using the Kolmogorov-Smirnov test. Data are presented as mean ± SD, except for the number and percentage of boys and girls with MetS. The chi-square test was used to evaluate differences in the prevalence of MetS (%) between boys and girls. Mean differences between boys and girls were assessed using Student’s t-test. Odds ratios between various atherogenic parameters and MetS were calculated using logistic regression analysis with three models: Model 1, unadjusted; Model 2, adjusted for age; and Model 3, adjusted for age, systolic and diastolic blood pressure, TG, body fat percentage, BMI, WC, HC, WHR, and birth weight. The goodness of fit for the logistic regression analyses was evaluated using the Hosmer-Lemeshow test. The area under the receiver operating characteristics (ROC) curves was calculated to assess the ability of the IR indices to discriminate IR. Cutoff points were proposed following the calculation of the Youden index (sensitivity + specificity – 1).

## Results

### Baseline characteristics of the participants

Table 1 describes the baseline characteristics of the participants. A total of 981 adolescents participated in our study, of whom 53.5% were girls. Boys had higher weight, WC, WHR, TG, and insulin concentrations than girls (p < 0.05). Conversely, girls had a higher body fat percentage compared to boys (p < 0.05).

**Table 1:** Characteristics of the participants.

	Boys (n = 456)		Girls (n = 525)	
	Mean	SD	Mean	SD
Age (y)	13.2	1.2	13.3	1.2
Weight (kg)	57.1	14.1	53.1	11.0***
Fat (%)	27.3	8.3	29.6	7.8*
BMI (kg/m^2^)	21.5	4.0	21.1	3.6
WC (cm)	73.7	11.8	71.3	9.6***
HC (cm)	84.2	9.9	83.8	8.6
WHR	0.88	0.06	0.87	0.06***
Glucose (mg/dL)	86.2	31.2	85.2	28.7
TG (mg/dL)	129.2	59.3	125.0	46.2*
Cholesterol (mg/dL)	175.8	35.6	174.6	33.2
LDL-c (mg/dL)	93.4	23.6	92.9	22.5
HDL-c (mg/dL)	40.1	2.8	40.0	3.1
SBP (mmHg)	119.6	15.7	116.9	15.1
DBP (mmHg)	64.5	9.2	63.9	8.8
Insulin (mU/L)	21.0	10.2	20.2	9.0*
HBA1c (%)	4.8	2.3	4.6	1.2
HOMA-IR	4.5	2.9	4.3	3.1
McAuley	5.6	0.6	5.7	0.5
QUICKI	0.31	0.16	0.31	0.15
G:I	4.6	2.0	4.6	1.8

BMI, body mass index; WC, waist circumference; HC, hip circumference; WHR, waist-to-hip ratio; TG, triglycerides; LDL-c, low-density lipoprotein cholesterol; HDL-c, high-density lipoprotein cholesterol; SBP, systolic blood pressure; DBP, diastolic blood pressure; HBA1c (%), glycated hemoglobin; HOMA-IR, homeostatic model assessment of insulin resistance; QUICKI, quantitative insulin sensitivity check index; G:I, glucose to insulin ratio. *, p < 0.05; ***, p < 0.001 vs boys.

### Insulin resistance indices among adolescents with normal-weight, overweight and obesity

Table 2 shows the mean values of the different IR indices assessed in our study among adolescents with normal-weight, overweight and obesity, which were categorized according to the WHO categorization [33]. Values of percentage of HBA1c, fasting insulin and HOMA-IR significantly increased; and McAuley,QUICKI and SPISE indices significantly decreased progressively from adolescents with normal-weight to those with obesity both in boys and girls. Differences between normal-weight and overweight and between overweight and obese were statistically significant in both groups. For the glucose to insulin ratio, significant differences were only observed between obese and normal-weight adolescents

**Table 2:** Comparison of insulin resistance indices and factors among adolescents with normal-weig.ht, overweight or obesity.

	Normal-weight		Overweight		Obesity	
	Mean	SD	Mean	SD	Mean	SD
**Boys (n)**	381		49		26	
HbA1c (%)	4.3	0.4	5.5	0.3***	11.1	7.0***^, ###^
Insulin	17.2	1.1	33.4	11.1***	53.2	5.2***^, ###^
HOMAIR	3.7	1.3	6.8	2.7***	12.2	5.7***^, ###^
McAuley	5.8	0.4	5.0	0.5***	4.2	0.4***^, ###^
QUICKI	0.32	0.01	0.29	0.02***	0.27	0.01***^, ###^
G:I	5.0	1.9	2.8	1.2	1.7	0.8***
SPISE	8.4	1.6	5.7	0.3***	4.5	0.6***^,##^
**Girls (n)**	452		49		24	
HbA1c(%)	4.3	0.3	5.5	0.3***	9.4	1.8***^, ###^
Insulin (mU/L)	17.2	1.2	32.5	11.3***	51.7	4.1***^, ###^
HOMAIR	3.6	1.2	6.9	3.8***	13.2	7.0***^, ###^
McAuley	5.8	0.3	5.0	0.6***	4.1	0.6***^, ###^
QUICKI	0.32	0.01	0.30	0.02***	0.27	0.01***^, ###^
G:I	4.9	1.7	2.9	1.1	2.0	0.9***
SPISE	8.5	1.5	5.7	0.4***	4.6	0.5***^,##^

HBA1c, glycated hemoglobin; HOMA-IR, homeostatic model assessment of insulin resistance; QUICKI, quantitative insulin sensitivity check index; G:I, glucose to insulin ratio; SPISE, single point insulin sensitivity estimator. ***, p < 0.001 vs normal-weight; ^###^, p < 0.001 vs overweight, ^##^, p < 0.01 vs overweight.

### Insulin resistance indices among adolescents with or without diabetes

Table 3 shows the mean values of the different IR indices assessed in our study among adolescents with or without diabetes. Adolescents were categorized according to the IDF criteria, considering adolescents with diabetes those with fasting blood glucose ≥ 126 mg/dL and HbA1c ≥ 6.5% [28]. Values for fasting blood glucose, fasting insulin, HOMA-IR and the glucose to insulin ratio were significantly higher in adolescents with diabetes in both genders in comparison to those without diabetes. Conversely, values for McAuley, QUICKI and SPISE indices were significantly lower in adolescents with diabetes in comparison to adolescents without diabetes.

**Table 3:** Comparison of insulin resistance indices and factors between adolescents with or without diabetes.

	Non-Diabetic		Diabetic	
	Mean	SD	Mean	SD
**Boys (n)**	406		50	
Glucose (mg/dL)	77.9	6.9	153.2	59.0***
HbA1c (%)	4.4	0.5	6.1	0.9***
Insulin (mU/L)	19.4	7.1	33.8	18.7***
HOMAIR	3.7	1.4	10.5	4.6***
McAuley	5.7	0.4	4.4	0.7***
QUICKI	0.32	0.01	0.28	0.01***
G:I	4.3	0.9	7.2	5.0***
SPISE	8.2	1.7	5.8	1.6***
**Girls (n)**	476		49	
Glucose (mg/dL)	78.0	7.1	155.1	55.0***
HbA1c(%)	4.4	0.5	6.7	3.0***
Insulin (mU/L)	18.8	6.2	33.2	17.9***
HOMAIR	3.6	1.2	11.0	6.0***
McAuley	5.8	0.3	4.6	0.9***
QUICKI	0.32	0.01	0.28	0.01***
G:I	4.4	0.8	7.1	4.8***
SPISE	8.2	1.6	6.3	2.0***

HBA1c, glycated haemoglobin; HOMA-IR, homeostatic model assessment of insulin resistance; QUICKI, quantitative insulin sensitivity check index; G:I, glucose to insulin ratio; SPISE, single point insulin sensitivity estimator. ***, p < 0.001 vs non-diabetic.

### Insulin resistance indices according to the presence of metabolic syndrome

Values of the IR indices according to the presence of MetS in adolescents are presented in Table 4. Significant higher values for HbA1c, fasting insulin and HOMA-IR were observed in adolescents with MetS of both genders in comparison to those without MetS. Conversely, values for McAuley, QUICKI, SPISEindices, and the glucose to insulin ratio were significantly lower in MetS-adolescents in comparison to non-MetS adolescents.

**Table 4:** Insulin resistance indices according to the presence of metabolic syndrome.

	Non-MetS		MetS	
	Mean	SD	Mean	SD
**Boys (n)**	422		34	
HbA1c (%)	4.4	0.8	9.3	6.7***
Insulin	18.8	6.3	47.8	11.1***
HOMAIR	4.0	2.0	10.7	5.1***
McAuley	5.7	0.5	4.4	0.3***
QUICKI	0.31	0.01	0.28	0.01***
G_I_ratio	4.8	1.9	2.2	1.7***
SPISE	8.1	1.7	4.8	0.8***
**Girls (n)**	428		97	
HbA1c (%)	4.2	0.3	6.2	2.0***
Insulin (mU/L)	17.2	1.9	33.4	14.5***
HOMAIR	3.6	1.6	7.5	5.4***
McAuley	5.8	0.3	5.0	0.8***
QUICKI	0.32	0.01	0.29	0.02***
G_I_ratio	5.0	1.7	3.1	1.6***
SPISE	8.5	1.5	5.8	1.0***

MetS, metabolic syndrome; HBA1c, glycated hemoglobin; HOMA-IR, homeostatic model assessment of insulin resistance; QUICKI, quantitative insulin sensitivity check index; G:I, glucose to insulin ratio; SPISE, single point insulin sensitivity estimator. ***,p < 0.001 vs Non-MetS. T test of independent samples.

### Regression Analyses Between Insulin Resistance Indices And HOMA-IR

Table 5 shows the results of the multivariate regression analyses for the IR indices analyzed indices. The McAuley and QUICKI indices negatively correlated with HOMA-IR in all three models in both boys and girls (p < 0.001). Conversely, SPISE index positively correlated with HOMA-IR in all three models in both boys and girls (p < 0.001). However, in none of the three models significant correlations were observed between HOMA-IR and the glucose to insulin ratio.

**Table 5:** Multivariate regression analyses for the relationship between various indexes related to insulin resistance and HOMA-IR.

	Model 1			Model 2			Model 3		
	β	SD	p	β	SD	p	β	SD	p
**Boys**									
McAuley	−4.161	0.118	<0.001	−4.162	0.118	<0.001	−4.941	0.281	<0.001
QUICKI	−165.998	3.249	<0.001	−165.934	3.254	<0.001	−170.594	6.362	<0.001
G:I	0.022	0.067	0.739	0.025	0.067	0.711	0.034	0.080	0.675
SPISE	1.356	0.129	<0.001	1.346	0.129	<0.001	0.792	0.204	<0.001
**Girls**									
McAuley	−4.865	0.121	<0.001	−4.865	0.121	<0.001	−5.153	0.298	<0.001
QUICKI	−178.909	3.726	0.021	−179.121	3.710	<0.001	−164.915	6.300	<0.001
G:I	0.000	0.073	0.998	0.001	0.073	0.992	0.015	0.069	0.827
SPISE	1.126	0.104	<0.001	1.126	0.104	<0.001	0.741	0.219	<0.001

Model 1: non-adjusted model.

Model 2: adjusted for age.

Model 3: adjusted for age, systolic and diastolic blood pressures, triglycerides, body fat percentage, body mass index, waist circumference, hip circumference, waist-to-hip ratio and weight at birth.

QUICKI, quantitative insulin sensitivity check index; G:I, glucose to insulin ratio; SD, standard deviation; SPISE, single point insulin sensitivity estimator.

### Receiver operating characteristic analysis of insulin resistance indices for Predicting insulin resistance

The results of the ROC analysis for the McAuley index, QUICKI index, SPISE index, and glucose to insulin ratio for predicting IR are shown in Table 6. The area under the curve (AUC) of ROC curves was the highest for the QUICKI index (1.00 ± 0.00) both in boys and girls. Good predictive values were also observed for the McAuley index (0.983 ± 0.004 in boys, and 0.977 ± 0.005 in girls) and for the SPISE index (0.896 ± 0.015 in boys, and 0.852 ± 0.017 in girls). Cut-off points for the McAuley and QUICKI indices were 5.794 and 0.316 for both boys and girls. In contrast, in both genders, glucose to insulin ratio exhibited a poor predictive ability for IR.

**Table 6:** AUC, optimal cutoff point, sensitivity, specificity, and Youden index in ROC analysis of different indexes for predicting insulin resistance.

	AUC	SD	95% CI	p	Cutoff	Sensitivity	Specificity	Youden Index
**Boys**								
McAuley	0.983	0.004	0.974-0.991	<0.001	5.794	0.900	0.983	0.883
QUICKI	1.000	0.000	1.000-1.000	<0.001	0.316	1.000	0.977	0.977
G:I	0.525	0.032	0.463-0.587	0.373	3.541	1.000	0.339	0.339
SPISE	0.896	0.015	0.867-0.926	<0.001	7.880	0.707	0.827	0.535
**Girls**								
McAuley	0.977	0.005	0.968-0.987	<0.001	5.794	0.896	0.980	0.876
QUICKI	1.000	0.000	1.000-1.000	<0.001	0.316	1.000	0.985	0.985
G:I	0.479	0.030	0.421-0.537	0.421	3.693	0.988	0.309	0.397
SPISE	0.852	0.017	0.819-0.885	<0.001	7.785	0.738	0.792	0.530

ROC, receiver operator characteristic; QUICKI, quantitative insulin sensitivity check index; G:I, glucose to insulin ratio; SPISE, single point insulin sensitivity estimator; AUC, area under the curve; SD, standard deviation; CI, confidence interval. Youden Index was calculated as sensitivity + specificity − 1.

## Discussion

The incidence of obesity in children and adolescents continues to rise globally [1]. This trend is associated with an increased likelihood of developing cardiovascular complications in adulthood, largely due to its close link with type 2 diabetes and IR during early life stages [2,3,5]. While numerous studies have evaluated the effectiveness of indirect methods for detecting IR in adult populations [15–18], there remains a insufficiency of data regarding their performance in adolescents. Our study aimed to address this gap by comparing the predictive accuracy of HOMA-IR, McAuley, QUICKI, SPISE indices, and the glucose-to-insulin ratio in a cohort of Spanish adolescents, while also determining practical cut-off values for IR prediction in this group.

Key findings revealed that male participants showed significantly higher weight, waist circumference (WC), waist-to-hip ratio (WHR), triglyceride (TG), and insulin levels compared to female participants, whereas females exhibited a greater percentage of body fat. These observations differ from the findings of Reckziegel et al. [34], in adolescents from Brazil, who reported elevated WC, TG, and insulin levels in females relative to males. Such disparities may be attributed to factors including ethnic differences and sample sizes. The greater adiposity seen in females aligns with known sexual dimorphism, which manifests during adolescence and is influenced by sex steroid hormones like estrogens. These hormones affect the central nervous system—particularly the hypothalamus—which plays a critical role in regulating energy balance and body weight through modulation of appetite and metabolism [35,36].

In relation to IR indices in participants with normal weight, overweight and obesity, in our study, HOMA-IR values increased significantly with BMI. These results are consistent with those described by Siqueira de Andrade et al. [37], in their study with Brazilian adolescents. who found higher HOMA-IR values among adolescents with excess weight. According to Shashaj et al. [11], HOMA-IR values are useful clinical practice and are accurate for the suspicion of the occurrence of cardiometabolic risk factors in adolescents, such as abnormal serum lipid and glucose levels, which are related to MetS. On the other hand, the values of the McAuley and QUICKI indices decreased significantly and progressively according to BMI in both boys and girls. These results are consistent with the findings described by Garg et al. [38], in their study with urban Indian adolescents. Likewise, a similar pattern of McAuley index has been reported in adolescents from New Zealand [39]. Similar values of McAuley index have also been found in adult populations from Pakistan and Sri Lanka [40,41]. The values of the SPISE index also decreased significantly and progressively in both boys and girls as BMI increased, which aligns with the findings reported by Tantari et al. [42] in Italian adolescents with overweight or obesity. For the glucose to insulin ratio, only significant differences were observed among participants with obesity and normal-weight, findings that are consistent with those described by Pizano-Zárate et al. [43], in their study with Mexican adolescents.

When examining parameters linked to glucose homeostasis, both adolescents with MetS or diabetes displayed elevated HbA1c, fasting insulin, and HOMA-IR levels compared to their peers without MetS or diabetes. These findings concur with a systematic review of an Iranian population by Zahedi et al. [44], which highlighted positive associations of fasting insulin, HOMA-IR, and HbA1c with MetS. In contrast, McAuley, QUICKI, and SPISE indices were significantly lower among adolescents with MetS or diabetes.

ROC curve analysis revealed that McAuley,QUICKI, and SPISEindices had superior discriminatory power for IR, while the glucose-to-insulin ratio performed poorly. The QUICKI index yielded the highest area under the curve (AUC) in both sexes (1.000), followed by the McAuley index (0.983 in males and 0.977 in females), and SPISE index (0.896 in males and 0.852 in females), respectively. These results stand in contrast to findings from Anoop et al. [45] in a southern Indian population, which reported much lower AUCs for QUICKI (0.260) and McAuley (0.180). Similarly, Kim et al. [46], found a McAuley index AUC of 0.85 with a cut-off of 5.3 in Korean subjects, slightly below the values observed in our study. However, Kaiser et al. [47] in a study conducted in Arab adolescents, and another one conducted by Son et al. [48] in Korean Adolescents, reported AUC values (0.853 and 0.961, respectively) for SPISE index similar than ours. These findings underscore the potential role of serum lipid metabolism, especially triglycerides, as a key contributor to early IR. Elevated TG levels, resulting from hyperinsulinemia, may induce pancreatic beta-cell dysfunction through intracellular lipid accumulation [49]. Kim et al. [46] also proposed that such metabolic cascades can perpetuate a vicious cycle worsening insulin regulation. Our data support the notion that dyslipidemia measurement may serve as a valuable indicator of early metabolic dysfunction, even in individuals with preserved hepatic glucose uptake. On the other hand, the glucose-to-insulin ratio showed limited predictive capacity for IR in both sexes.

Taken together, our findings suggest that QUICKI, McAuley, and SPISEindices could serve as reliable surrogate markers for screening both IR risk and metabolic syndrome in Spanish adolescents. However, as noted by Antuna-Puente et al. [50], these indices require further validation in larger cohorts encompassing a broad spectrum of insulin sensitivity and glucose tolerance before being routinely implemented in clinical practice.

The present study has noteworthy strengths and some limitations. First of all, we studied a large cohort representing a wide range of age of both sexes, contributing to obtaining robust results. Additionally, the studied sample can be considered homogeneous, as participating children and adolescents were from the same geographical region, and shared similar culture, lifestyle, and eating habits. On the other hand, the cross-sectional design of the study made it impossible for us to explore the causal relationship between the studied variables. Furthermore, the absence of information regarding the puberty status of the participants due to a lack of authorization for its collection requires caution when interpreting the results.

In conclusion, although similar results with other authors were observed in the logistic regression analyses, discrepancies were noticed in the analyses for assessing the predictive abilities of these indices in comparison to other authors. However, QUICKI,McAuley, and SPISE indices revealed as useful indicators with a strong capacity to predict IR in the Spanish adolescent sample studied, regardless of gender. Taking into account our results, although new studies with large cohorts of subjects are necessary to obtain more solid results, McAuley, QUICKI, and SPISEindices have potential as new indirect methods to be used in clinical practice by health professionals in the early detection not only of IR, but also of related long-term metabolic and cardiovascular complications.
